# Combined obstructive airflow limitation associated with interstitial lung diseases (O-ILD): the bad phenotype ?

**DOI:** 10.1186/s12931-022-02006-9

**Published:** 2022-04-11

**Authors:** Julien Guiot, Monique Henket, Anne-Noëlle Frix, Fanny Gester, Marie Thys, Laurie Giltay, Colin Desir, Catherine Moermans, Makon-Sébastien Njock, Paul Meunier, Jean-Louis Corhay, Renaud Louis

**Affiliations:** 1 Respiratory Department of CHU Liège, Domaine Universitaire du Sart-Tilman, B35, 4000 Liege, Belgium; 2 Medico-Economic and Data Department of CHU Liège, Liege, Belgium; 3 Radiology Department of CHU Liège, Liege, Belgium

**Keywords:** Interstitial lung disease, Progressive fibrosing ILD, Idiopathic pulmonary fibrosis, Obstructive lung disease

## Abstract

**Background:**

Patients suffering from combined obstructive and interstitial lung disease (O-ILD) represent a pathological entity which still has to be well clinically described. The aim of this descriptive and explorative study was to describe the phenotype and functional characteristics of a cohort of patients suffering from functional obstruction in a population of ILD patients in order to raise the need of dedicated prospective observational studies and the evaluation of the impact of anti-fibrotic therapies.

**Methods:**

The current authors conducted a retrospective study including 557 ILD patients, with either obstructive (O-ILD, n = 82) or non-obstructive (non O-ILD, n = 475) pattern. Patients included were mainly males (54%) with a mean age of 62 years.

**Results:**

Patients with O-ILD exhibited a characteristic functional profile with reduced percent predicted forced expired volume in 1 s (FEV1) [65% (53–77) vs 83% (71–96), p < 0.00001], small airway involvement assessed by maximum expiratory flow (MEF) 25/75 [29% (20–41) vs 81% (64–108), p < 0.00001], reduced sGaw [60% (42–75) vs 87% (59–119), p < 0.01] and sub-normal functional residual capacity (FRC) [113% (93–134) vs 92% (75–109), p < 0.00001] with no impaired of carbon monoxide diffusing capacity of the lung (DLCO) compared to those without obstruction. Total lung capacity (TLC) was increased in O-ILD patients [93% (82–107) vs 79% (69–91), p < 0.00001]. Of interest, DLCO sharply dropped in O-ILD patients over a 5-year follow-up. We did not identify a significant increase in mortality in patients with O-ILD. Interestingly, the global mortality was increased in the specific sub-group of patients with O-ILD and no progressive fibrosing ILD phenotype and in those with connective tissue disease associated ILD especially in case of rheumatoid arthritis.

**Conclusions:**

The authors individualized a specific functional-based pattern of ILD patients with obstructive lung disease, who are at risk of increased mortality and rapid DLCO decline over time. As classically those patients are excluded from clinical trials, a dedicated prospective study would be of interest in order to define more precisely treatment response of those patients.

**Supplementary Information:**

The online version contains supplementary material available at 10.1186/s12931-022-02006-9.

## Background

Interstitial lung disease (ILD) is known to be a heterogeneous group of diseases characterized by irreversible fibrotic changes in the lung parenchyma [1–3]. The archetypal disease of ILD is idiopathic pulmonary fibrosis (IPF) which is known to be progressive over the time despite specific anti-fibrotic therapies [4, 5]. In IPF, even though specific association with genetic susceptibility have been described, it is widely admitted that smoking status is a risk factor for developing IPF [6]. However, other subtypes can also exhibit progressive phenotypes including connective tissue disease-associated ILD (CTD-ILD) [7–9], fibrotic hypersensitivity pneumonitis (HP) [10], idiopathic non specific interstitial pneumonia (iNSIP) [11, 12], organizing pneumonia [13], unclassifiable ILD, rarely sarcoidosis [14, 15], and ILD associated with occupational exposures [16, 17].

Although ILDs display a phenotype characterized by fibrotic lesions in lung leading to a reduction in lung capacity, some of them can also be associated with a specific bronchial disease. Moreover, a subset of IPF patients presenting extended emphysematous lesions, named combined pulmonary fibrosis and emphysema (CPFE), are associated with more severe outcome [18–21]. Both IPF and CPFE patients may develop acute exacerbations that have important implications for the treatment and prognosis of the disease [20]. In addition, Kitaguchi et al. have evaluated differences in pulmonary function test between CPFE patients with and without airflow obstruction [22]. They found that the proportions of emphysema and pulmonary fibrosis on chest High-resolution computed tomography (HRCT) were different between CPFE patients with and without airflow obstruction.

Some ILDs can manifest themselves both at a bronchial and parenchymal level. For instance, sarcoidosis which is recognized to be a granulomatous disease, is frequently associated with airway obstruction, mimicking asthma or COPD symptoms, and more severe cases induces decline of lung function [(reduction of Forced expired Volume in 1s (FEV1)] [23]. Indeed, sarcoidosis can affect airway at any level resulting in a significant airway obstruction in up to 4–63% of cases [24]. Inhalation of tobacco smoke is one of the common risk factor in ILD [6, 25] and COPD [26, 27] that can lead combined pathological situations.

COPD is defined as a preventable and treatable disease that is characterized by persistent respiratory symptoms and airflow limitation that is due to airway and/or alveolar abnormalities associated with a persistent airway obstruction [28–30]. It can be challenging to identify obstructive airflow limitation in patients suffering from ILDs due to the reduction of total lung capacity (TLC). Whereas HRCT is nowadays the major way to explore and diagnose ILDs [31, 32], it is of low interest in order to identify O-ILD patients. Facing a significant vital forced capacity (FVC) reduction in the ILDs, this can, by definition, reduce the capability of clinicians to confirm obstructive airflow limitation. Moreover, emphysematous hyperinflation can lead to an overestimation of the total lung capacity (TLC) than can underestimate ILD severity.

In most clinical trials evaluating anti-fibrotic therapies, ILD patients exhibiting significant airway obstruction are systematically excluded, reducing the validation of anti-fibrotic treatments for this subset of patients. Therefore, there is an unmet clinical need to better define the subset of O-ILD patients and subsequently investigate the use of ILD-targeted therapies (such as antifibrotics) in this group.

The aim of this descriptive and explorative study was to describe the phenotype and functional characteristics of O-ILD patients. For this, we retrospectively analyzed our clinical database from Liège University Hospital in order to define the subgroup of patients with ILD exhibiting airway obstruction.

## Methods

### Patient cohorts

A retrospective observational cohort study on patients suffering from ILD recruited from our ambulatory care policlinic at the University Hospital of Liège from April 31st 2004 to January 28th 2021 in our tertiary reference center was performed. We identified patients using electronic hospital records and assessed them for eligibility. We included patients aged from 18 years old and over. All cases were discussed in a multidisciplinary group about ILDs composed of a pulmonologist, a specialist in pulmonary rehabilitation, a rheumatologist, a radiologist, a pathologist and a specialist in occupational medicine [33]. Prior to the analysis, ILDs were classified in five different sub-groups: IPF (n = 68), non IPF-idiopathic interstitial pneumonia (IIP) (n = 91), CTD-ILD (n = 186), sarcoidosis (n = 149) and other-ILDs (n = 63).

Airway obstruction was defined by a Tiffeneau index (FEV1/FVC assessed after having treated patients with bronchodilator salbutamol 400 µg) < 70%. The demographic parameters collected are those evaluated on the day of the diagnosis. The protocol was approved by the ethics committee of CHU of Liège (Belgian number: B707201422832; ref: 2014/302).

### Collected data

Information was collected on patients’ characteristics (age, gender, smoking status) and clinical characteristics (diagnosis, medical history, radiological patterns, lung function, biomarkers, treatment). Data on medications were collected throughout the study, including immunosuppressive agents.

Peripheral blood cell count has been studied in order to identify mainly patient with hypereosinophilic status. Moreover, monocyte blood count has been reported to be associated with worse outcome in patients suffering from ILD justifying the need of evaluating those parameters [34].

### Statistical analysis

Statistical analysis were performed using TIBCO Statistica (13.3.1) software. A p-value of less than 0.05 was considered to be statistically significant. Descriptive analyses of patient characteristics were completed using standard summary statistics for distributions. Results are expressed as frequency tables for qualitative variables and as median and interquartile range (IQR) for continuous variables. Contingency tables were analyzed by two-tailed chi-square test, and two-tailed Mann Whitney test was used to compare continuous variable, followed by Bonferroni correction for multiple comparisons. Overall, survival was represented by a Kaplan–Meier curve. Survival between groups was compared by Log Rank test and the Hazard Ratio (Mantel–Haenszel) was reported. For the longitudinal study, the evolution of parameters between two visits was analyzed with a repeated measures using two-tailed ANOVA test followed by the post hoc Tukey’s test.

## Results

### Subject demographic

**Fig. 1:**
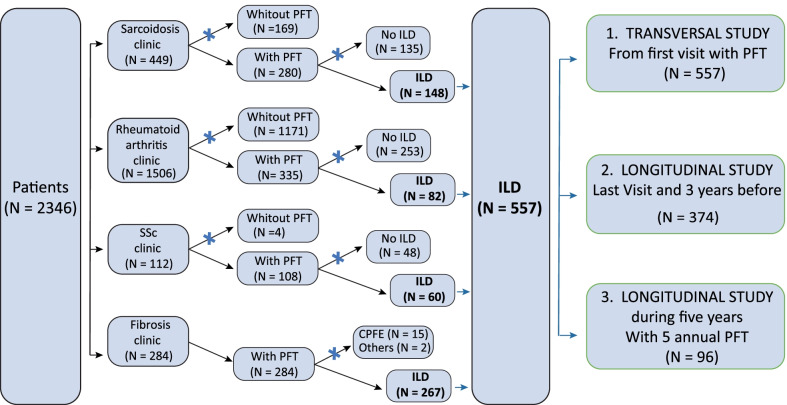
Flow chart of patient inclusion in our study

We retrospectively recruited 557 ILD patients from our ambulatory care policlinic at the University Hospital of Liège (Fig. 1). We identified a proportion of 14.7% (n = 82) of patients suffering from O-ILD whereas 35% of the entire cohort exhibit a progressive fibrosing-ILD (PF-ILD) pattern according to the INBUILD criteria [35]. Demographic characteristics of the subjects are shown in Table 1 and Additional file 1: Table S1. The median age of ILD patients is 62 (50–71) years old with a male predominancy (54%). In non O-ILD subgroup, there are 13.3% of IPF, 16.6% of IIP, 34.5% of CTD-PF, 25.3% of sarcoidosis and 10.3% of other ILDs, whereas in O-ILD subgroup, there are 6.1% of IPF, 14.6% of IIP, 26.8% of CTD-PF, 35.4% of sarcoidosis and 17.1% of other ILDs. In the O-ILD cohort, we did not identify significant differences in comparison to the non O-ILD group for smoking status, age or gender. Of interest, O-ILD patients exhibited a lower BMI compared to patients with non-obstructive pattern.

**Table 1:** Patient characteristics

	Non O-ILD n = 475 (85.3%)	O-ILD n = 82 (14.7%)	p-value
Demography			
Age (years)	62 (50–71)	61 (49–70)	n.s
Gender (M/F)	252/223	51/31	n.s
BMI (Kg/m^2^)	26 (24–30)	25 (22–28)	** < 0.01**
Smokers NS/FS/CS(%)	44%/44%/13%	31%/57%/12%	n.s
ILD category			
IPF	63 (13.3%)	5 (6.1%)	
IIP	79 (16.6%)	12 (14.6%)	
CTD-ILD	164 (34.5%)	22 (26.8%)	
Sarcoidosis	120 (25.3%)	29 (35.4%)	
Other ILD	49 (10.3%)	14 (17.1%)	

Bold to indicate statistically significant* p* values

Data are expressed as median (IQR)

*BMI* body mass index, *CTD-ILD* connective tissue disease-associated ILD, *FS* former smoker, *ILD* interstitial lung disease, *IIP* idiopathic interstitial pneumonia, *IPF* idiopathic pulmonary fibrosis, *n.s.* not significant, *NS* non smoker, *non O-ILD* non obstructive-interstitial lung disease, *O-ILD* obstructive-interstitial lung disease, *S* smoker

### Blood analysis

The results of blood leukocyte counts are listed in Table 2. Patients with O-ILD presented a higher concentration of blood leucocytes compared to the non O-ILD cohort, with an elevated level of neutrophils (5.48 cell/mm^3^ (4.29–8.33) vs 4.94 cell/mm^3^ (3.67–6.65), p < 0.05). Of note, we did not identify significant differences for monocyte count and classical inflammatory markers (CRP and fibrinogen).

**Table 2:** Blood characteristics

	Non O-ILD (n = 475)	O-ILD (n = 82)	p-value
Blood analysis			
Leucocyte (10^3^/µl)	7.99 (6.07–9.86)	8.24 (6.68–11.20)	n.s
Neutrophil %	66 (57–74)	71 (63–76)	** < 0.05**
Cell/mm^3^	4.94 (3.67–6.65)	5.48 (4.29–8.33)	** < 0.05**
Lymphocyte %	22 (15–29)	19 (12–24)	** < 0.05**
Cell/mm^3^	1.59 (1.16–2.17)	1.54 (0.94–2.17)	n.s
Monocyte %	7.6 (5.9–9.8)	7.5 (5.1–9.8)	n.s
Cell/mm^3^	0.60 (0.44–0.78)	0.60 (0.44–0.80)	n.s
Eosinophil %	2.3 (1.3–3.75)	1.9 (0.6–3.7)	n.s
Cell/mm^3^	0.17 (0.09–0.29)	0.16 (0.08–0.27)	n.s
Basophil %	0.5 (0.2–0.7)	0.4 (0.2–0.7)	n.s
Cell/mm^3^	0.03 (0.02–0.05)	0.03 (0.02–0.05)	n.s
CRP (mg/L)	4.83 (1.97–17.10)	9.35 (2.68–27.47)	n.s
Fibrinogen (g/L)	3.84 (3.15–4.93)	3.78 (3.33–5.78)	n.s

Bold to indicate statistically significant* p* values

*CRP* C-reactive protein, *non O-ILD* non obstructive-interstitial lung disease, *O-ILD* obstructive-interstitial lung disease

### Pulmonary functional tests

Pulmonary functional tests are showed in Table 3. Spirometric values were significantly different between O-ILD patients and those without obstruction. O-ILD patients exhibited a higher levels of TLC (93% (82–107) vs 79% (69–91), p < 0.00001) and functional residual capacity (FRC) (113% (93–134) vs 92% (75–109), p < 0.00001) compared to those without O-ILD pattern. Of interest, there was no difference with regards to the FVC value but a significant reduction of the FEV1 in the O-ILD group compared to the non O-ILD group (65% (53–77) vs 83% (71–96), p < 0.00001). By definition, all patients in the O-ILD group were exhibiting a Tiffeneau index (FEV1/FVC) under 70%. As commonly identified in other obstructive airway diseases, O-ILD patients presented a significant reduction in their specific conductance as referred by the sGaw value compared to non O-ILD patients (60% (42–75) vs 87% (59–119), p < 0.01), and small airway involvement assessed by maximum expiratory flow (MEF) 25/75 (29% (20–41) vs 81% (64–108), p < 0.00001). DLCO and KCO were similar in both groups.

**Table 3:** Pulmonary functional tests

	Non O-ILD (n = 475)	O-ILD (n = 82)	p-value
Pulmonary functional test			
FEV1 (L)	2.31 (1.76–2.88)	1.75 (1.38–2.41)	** < 0.0001**
FEV1 (%pred.)	83 (71–96)	65 (53–77)	** < 0.0001**
FVC (L)	2.81 (2.16–3.64)	2.99 (2.32–3.89)	n.s
FVC (%pred.)	83 (69–96)	84 (72–98)	n.s
FEV1/FVCpost-BD	81 (77–86)	62 (58–67)	** < 0.0001**
MEF25/75 (L)	2.49 (1.87–3.17)	1.02 (0.60–1.36)	** < 0.0001**
MEF25/75 (%)	81 (64–108)	29 (20–41)	** < 0.0001**
RV (L)	1.76 (1.35–2.21)	2.43 (1.81–3.26)	** < 0.0001**
RV (%pred.)	83 (63–104)	116 (84–152)	** < 0.0001**
TLC (L)	4.73 (3.82–5.54)	5.78 (4.77–6.56)	** < 0.0001**
TLC (%pred.)	79 (69–91)	93 (82–107)	** < 0.0001**
DLco (mmol/kPa min)	4.67 (3.3–6.43)	4.94 (3.27–6.30)	n.s
DLco (%pred.)	56 (41–67)	58 (41–67)	n.s
KCO (mmol/kPa min/L)	1.18 (0.95–1.43)	1.09 (0.92–1.38)	n.s
KCO (%pred.)	82 (68–97)	77 (65–92)	n.s
FRC (L)	2.76 (2.33–3.56)	3.46 (2.94–4.64)	** < 0.0001**
FRC (%pred.)	92 (75–109)	113 (93–134)	** < 0.0001**
sGaw (L)^a^	1.15 (0.80–1.54)	0.69 (0.51–0.98)	** < 0.0001**
sGaw (%)^b^	87 (59–119)	60 (42–75)	** < 0.01**

Bold to indicate statistically significant* p* values

Data are expressed as median (IQR). Values not available

*DLCO* diffusing lung capacity of CO, *FEV1* forced expired volume in 1s, *FRC* functional residual capacity, *FVC* forced vital capacity, *ILD* interstitial lung disease, *KCO* DLCO/Alveola ventilation, *MEF* maximum expiratory flow, *NS/FS/CS* non smokers/former smokers/current smokers, *O-* obstructive-, *RV* residual volume, *TLC* total lung capacity, *sGaw* specific conductances

^a^n = 396 ILD et 67 O-ILD

^b^n = 208 ILD et 17 O-ILD

### Treatment characteristics

In the O-ILD group, we identified that 43% of patients were treated with bronchodilator therapies with a homogenous repartition between long-acting B2-agonists (LABA) and long-acting muscarinic antagonist (LAMA) (Table 4). Of note, 24% of them were treated with inhaled corticosteroids (ICS) and 21% of them with immunosuppressive therapies.

**Table 4:** Treatment characteristics

	Non O-ILD	O-ILD	
	(n = 475)	(n = 82)	p-value
Treatment, yes (%)			
Bronchodilator	45 (9%)	35 (43%)	** < 0.0001**
SABA	18 (4%)	13 (16%)	** < 0.0001**
LABA	32 (7%)	18 (22%)	** < 0.0001**
LAMA	1 (0%)	17 (21%)	** < 0.0001**
ICS	40 (8%)	20 (24%)	** < 0.0001**
OCS	98 (21%)	13 (16%)	
Immunosuppressive therapy	76 (16%)	17 (21%)	

Bold to indicate statistically significant* p* values

Among non O-ILD patients, 31 (7%) received a double therapy LABA/ICS. Among O-ILD patients, 9 (11%) received a double therapy LABA/ICS, 2 (2%) a double therapy LABA /LAMA and 7 (8%) a triple therapy (LABA/LAMA/ICS)

*LABA* long-acting B2-agonists, *LAMA* long-acting muscarinic antagonist, *ICS* inhaled corticosteroids, *ILD* interstitial lung disease, *O-ILD* obstructive-interstitial lung disease, *OCS* oral corticosteroids, *SABA* short acting B2 agonists

### Survival analysis

**Fig. 2:**
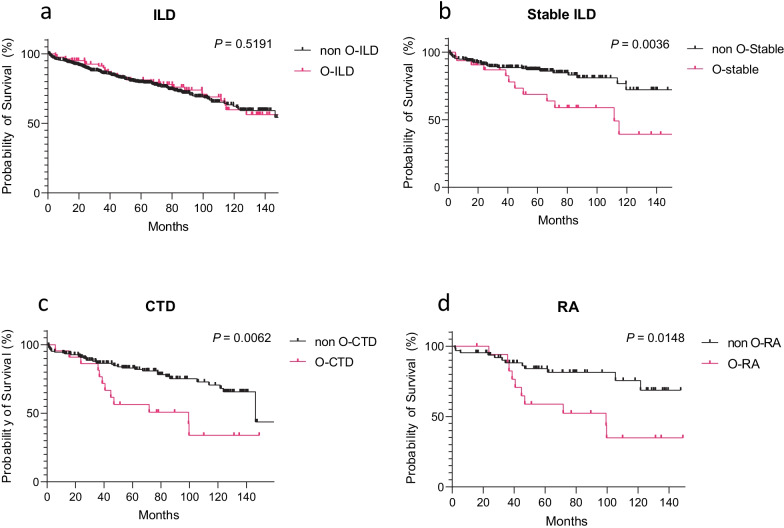
Survival of patients based on their O-ILD phenotype. Kaplan-Meyer curve comparing ILD with or without obstructive syndrome in **a** Global ILD cohort. **b** ILD patient without progressive ILD. **c** Connective tissue disease (CTD) associated pulmonary fibrosis (CTD-PF) cohort. **d** Rheumatoid arthritis (RA) associated pulmonary fibrosis cohort

In the global cohort, we did not identify any significant difference between patients with or without O-ILD (Fig. 2a; Table 5). Nevertheless, after having specifically studied the patients based on the initial ILD diagnosis, we found that patients with stable ILD (no PF-ILD phenotype) exhibiting an O-ILD pattern were displaying a reduced survival rate (111.5 months vs undefined median survival; p < 0.01) (Fig. 2b; Table 5). In the IPF cohort, we didn’t identify any significant differences between patient with or without the O-ILD phenotype (45 vs 33 months of median survival respectively, p > 0.05).

**Table 5:** Survival of O-ILD patients

		Median survival (months)			O-ILD/ILD
		Non O-ILD	O-ILD	Log rank *T test*	Hazard ratio (95% CI of ratio)
Figure 2a	Global ILD cohort	Undefined	156.9	n.s	0.9854 (0.6407–1.516)
Figure 2b	ILD patient without PF-ILD	Undefined	111.5	***p* = *0.0036***	3.529 (1.511–8.241)
Figure 2c	CTD cohort	146.4	99.4	***p* = *0.0062***	3.362 (1.411–8.011)
Figure 2d	RA cohort	Undefined	99.4	***p* = *0.0148***	3.413 (1.272–9.161)

Bold to indicate statistically significant* p* values

Kaplan-Meyer survival analysis of O-ILD patients in each cohort

*non O-ILD* non obstructive-interstitial lung disease, *O-ILD* bstructive-interstitial lung disease

In the CTD cohort, we interestingly found a significant reduced survival rate in patient suffering from the O-ILD pattern, with a median survival rate of 99 versus 146 months (p < 0.01) (Fig. 2c; Table 5).

### Pulmonary functional longitudinal follow-up

**Fig. 3:**
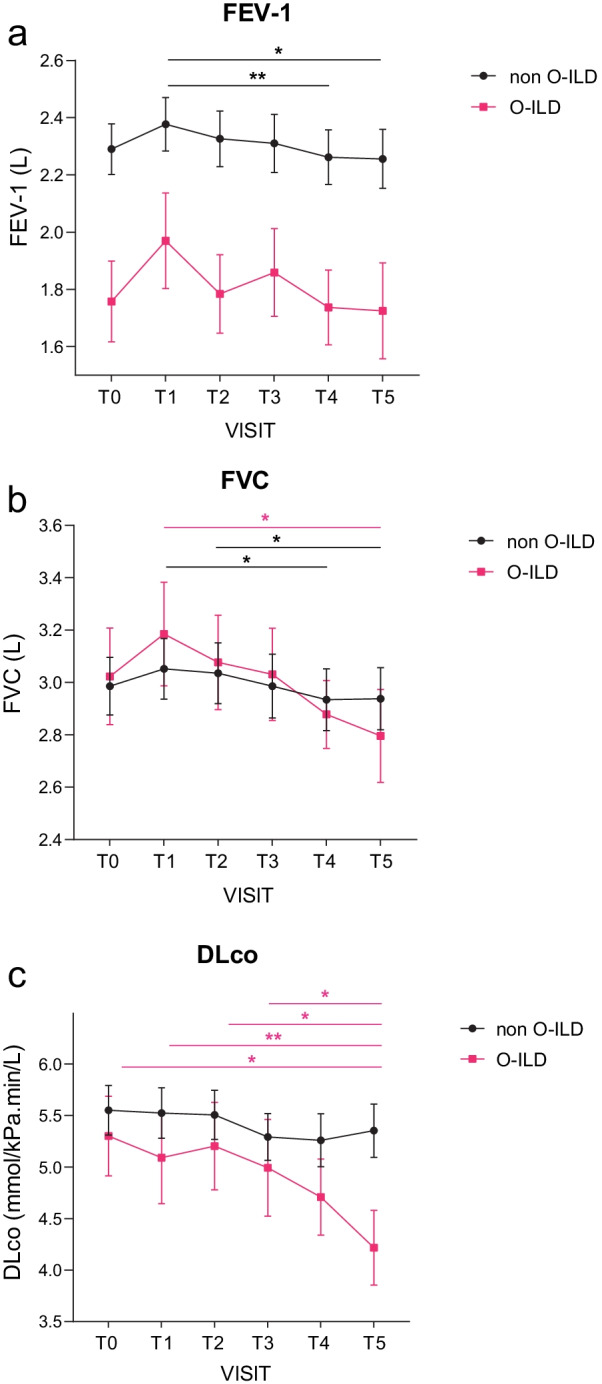
Pulmonary function tests (PFT) longitudinal follow-up over 5 years. Mean value of pulmonary function test over the 5 year follow-up comparing patients suffering from the obstructive phenotype (in red) and those without obstruction (in black). **a** Evolution of FEV-1 over 5 years. **b** Evolution of FVC over 5 years. **c** Evolution of DLCO over 5 years. Results are expressed with mean ± SEM *P < 0.05 **P < 0.01

In both cohorts, we identified a significant decrease in lung volumes as well as in DLCO. We didn’t find any significant differences between patients suffering from O-ILD and those without (Additional file 3: Table S3). We then sub-selected ILD patients (n = 96) with a full annual longitudinal follow-up of 5 years (non O-ILD group (n = 76): 37 CTD-PF, 6 IIP, 6 IPF, 25 sarcoidosis and 2 others; O-ILD group (n = 20): 4 CTD-PF, 1 IIP, 2 IPF, 10 sarcoidosis and 3 others) and re-analyzed the lung function decline. Interestingly, we found that DLCO was more severely decreased over the time in the O-ILD group (p < 0.05) (Fig. 3).

## Discussion

The current study provides a comprehensive analysis of a group of 577 patients suffering from ILD and retrospectively analyzed the evolution of patients suffering from ILD exhibiting a specific obstructive functional pattern (O-ILD). Even if ILD patients are typically presenting restrictive lung diseases, we cannot exclude that some of them can experience a mixed obstructive and restrictive disease, combined in the most severe cases with emphysema, known to be of bad prognosis in the particular case of IPF (CPFE) [5, 19].

In our study, we identified that around 15% of them were presenting a functional obstructive lung disease. Those O-ILD patients were displaying higher TLC with globally reduced FEV-1, specific conductance (sGaw) and increased FRC. Of interest, the longitudinal follow-up of lung function (FVC, FEV1 and DLCO) over 5 years didn’t show any significant difference between groups. Focusing on the mortality, obstructive patients with non-progressive ILD (based on the INBUILD criteria) were showing an increased mortality rate compared to those without obstruction [35]. Similarly, in CTD and particularly in RA, we identified a significant increase in mortality in patients with obstructive pattern. Conversely in IPF, we didn’t find any significant differences between the two groups.

To the best of our knowledge, this study is the first one exploring obstructive pattern in the particular field of ILD. Indeed, in clinical trial, patients with O-ILD are systematically excluded. As a result, few is known about the evolution of those patients and particularly for the response to new specific anti-fibrotic therapies. Based on our results, we can hypothesize that up to 15% of the patients are potentially considered as not able to participate. Even though the clinical evolution of those patients is generally considered as different, we didn’t show any significant differences between patients with or without obstruction with regards to the evolution of PFT over 3 years of follow-up. If obstruction pattern might have been caused by past smoking history, it worth noting the current smokers were equally distributed among ILD and O-ILD. It may explain why O-ILD did not have an increased FEV1 decline as opposed to ILD. It is also worth noting that O-ILD had greater blood neutrophil count while being less often treated with oral corticosteroids. The reason of this raised neutrophil count is unclear and its possible contribution to airway obstruction needs to be clarified [21, 36]. Of interest, the sub-analysis of the 5-year longitudinal follow-up identified that DLCO was decreasing more severely in the O-ILD cohort. Contrarily to what is seen for PFTs, we identified a significant increase in mortality in the obstructive group for non-PF-ILD patients and CTD-PF patients. Moreover, the overall evolution for IPF patients is similar in both groups.

In the particular case of sarcoidosis, several studies indicate that an obstructive pattern is common (4–67% of patients) while a mixed obstructive-restrictive disease is found in around 2–19% of them [24, 37]. The evidence of small airway obstruction originates from pathological studies identifying peribronchial lesions detectable in the HRCT examination by specific bronchial dilatations, mosaic parenchymal attenuation and air trapping [38]. Of note, sarcoidosis associated with airway obstruction is commonly exhibiting a higher morbi-mortality rate [39]. We didn’t find that this observation was specifically true in the particular case of patient with ILD associated to their sarcoidosis, leading us to hypothesize that in this specific case, the ILD is the leading cause of death.

The specific case of RA-ILD is of interest. Indeed, cigarette smoking may play a role in inducing antibody formation and has been linked to occurrence of anti-CCP antibodies induced by the promotion of lung proteins citrullination. Out of the conventional obstruction found similarly to what is seen in COPD, RA patients can suffer from follicular bronchiolitis and obliterative bronchiolitis [40]. Tobacco smoking is associated with an increased risk of RA-ILD (odds ratio of 3.8) for those who smoked > 25 pack-years) [41]. In our cohort, we found that 69% of the O-ILD patients were smokers or former smokers. In this context, we think that obstructive functional pattern in patients suffering from RA needs to be carefully assessed and particularly those with RA associated ILD. We also have to consider that patients suffering from RA-ILD could experience bronchiolitis that can mimic COPD experiencing a different outcome in this context. This point has to be address in further larger clinical trials.

Therapeutic options in O-ILD are limited, considering the lack of well-designed prospective clinical trials in order to confirm the potential benefit of both inhaled and oral anti-fibrotic therapies. Tobacco smoking should be discontinued. It is also surprising that only a small proportion of patients with O-ILD are treated with long-acting bronchodilator therapy (LAMA, LABA or LAMA-LABA) and ICS even though patients are symptomatic with a median FEV1 of 65%. It is therefore important in the follow-up of our patients with ILD to be vigilant to not underdiagnosed and undertreated O-ILD patients while we acknowledge the lack of dedicated clinical trial in order to guide clinicians.

## Conclusion

In conclusion, patients with combined obstructive and interstitial lung diseases exhibit a characteristic functional profile with reduced FEV-1, small airway involvement, a reduced sGaw value and sub-normal FRC with similar carbon monoxide diffusing capacity of the lung compared to those without obstruction. O-ILD patients can have normal total lung capacity, which may be responsible for its under recognition compared to the other ILDs. O-ILD patients exhibit a specific evolution over the time that underlies the need of dedicated clinical follow-up.

Our study identified that patients with O-ILD exhibit similar clinical lung function evolution over 5-years in case of IPF. By opposition, we found an increase in mortality in non-progressive ILD raising the hypothesis that the obstructive airflow limitation due to the bronchial involvement of the lung disease (asthma, COPD) can drive the long-term outcome of those patients. In the particular case of CTD-ILD, we also specifically found a strong increase in mortality associated to the obstructive lung pattern, and particularly in case of RA-ILD. The discrepancy between PFT and survival could be due to the methodology of the study, whereas DLCO progression in the O-ILD subgroup may be due to the fact that those patients survived longer than those in the non O-ILD subgroup, which induces survival biais.

Therefore, patients with O-ILD have to be considered for specific anti-fibrotic therapies in line with the similar clinical outcome found compared to IPF and PF-ILD without obstructive pattern. Patients suffering from CTD-PF require a close follow-up in order to reduce the increase overall mortality in this condition.

## Supplementary Information

**Additional file 1: Table S1.** Evaluation of demographic and blood characteristics in each disease.

**Additional file 2: Table S2.** Evaluation of pulmonary functional tests in each disease.

**Additional file 3: Table S3.** Evolution of pulmonary functional test over 3 years.

**Additional file 4: Fig. S1.** Evolution of pulmonary functional test over 3 years from the last visit.

## Data Availability

The data underlying this article are available in the article and in its online Additional Files. Further inquiries will be shared on reasonable request to the corresponding author.

## Abbreviations

BMI: Body mass index
CPFE: Combined pulmonary fibrosis and emphysema
CRP: C-reactive protein
CTD: Connective tissue disease
DLCO: Diffusing lung capacity of CO
FEV1: Forced expired volume in 1s
FRC: Functional residual capacity
FVC: Forced vital capacity
GFR: Glomerular filtration rate
HP: Hypersensitivity pneumonitis
ICS: Inhaled corticosteroids
IIP: Idiopathic interstitial pneumonia
ILD: Interstitial lung disease
IPF: Idiopathic pulmonary fibrosis
KCO: DLCO/Alveola ventilation
LABA: Long-acting B2-agonists
LAMA: Long-acting muscarinic antagonist
MEF: Maximum expiratory flow
NS/FS/CS: Non smokers/former smokers/current smokers
O-: Obstructive
OCS: Oral corticosteroids
PF-ILD: Progressive fibrosing-ILD
SABA: Short acting B2 agonists

## References

[1] Raghu G, Richeldi L (2017). Current approaches to the management of idiopathic pulmonary fibrosis. Respir Med.

[2] Travis WD, Costabel U, Hansell DM, King TE, Lynch DA, Nicholson AG (2013). An official American Thoracic Society/European Respiratory Society statement: update of the international multidisciplinary classification of the idiopathic interstitial pneumonias. Am J Respir Crit Care Med.

[3] Wells AU, Hirani N (2008). Interstitial lung disease guideline. Thorax BMJ Publishing Group Ltd.

[4] Liu Y-M, Nepali K, Liou J-P (2017). Idiopathic pulmonary fibrosis: current status, recent progress, and emerging targets. J Med Chem.

[5] Guiot J, Duysinx B, Seidel L, Henket M, Gester F, Bonhomme O (2018). Clinical experience in idiopathic pulmonary fibrosis: a retrospective study. Acta Clin Belg.

[6] Baumgartner KB, Samet JM, Stidley CA, Colby TV, Waldron JA (1997). Cigarette smoking: a risk factor for idiopathic pulmonary fibrosis. Am J Respir Crit Care Med.

[7] Castelino FV, Varga J (2010). Interstitial lung disease in connective tissue diseases: evolving concepts of pathogenesis and management. Arthritis Res Ther.

[8] Shao T, Shi X, Yang S, Zhang W, Li X, Shu J (2021). Interstitial lung disease in connective tissue disease: a common lesion with heterogeneous mechanisms and treatment considerations. Front Immunol.

[9] Nicholson AG, Colby TV, Wells AU (2002). Histopathological approach to patterns of interstitial pneumonia in patient with connective tissue disorders. Sarcoidosis Vasc Diffuse Lung Dis.

[10] Raghu G, Remy-Jardin M, Ryerson CJ, Myers JL, Kreuter M, Vasakova M (2020). Diagnosis of hypersensitivity pneumonitis in adults. An Official ATS/JRS/ALAT Clinical Practice Guideline. Am J Respir Crit Care Med.

[11] Belloli EA, Beckford R, Hadley R, Flaherty KR (2016). Idiopathic non-specific interstitial pneumonia. Respirology.

[12] Katzenstein AL, Fiorelli RF (1994). Nonspecific interstitial pneumonia/fibrosis. Histologic features and clinical significance. Am J Surg Pathol.

[13] Epler GR, Colby TV, McLoud TC, Carrington CB, Gaensler EA (1985). Bronchiolitis obliterans organizing pneumonia. N Engl J Med.

[14] Chen ES, Moller DR (2008). Etiology of sarcoidosis. Clin Chest Med.

[15] Coultas DB, Zumwalt RE, Black WC, Sobonya RE (1994). The epidemiology of interstitial lung diseases. Am J Respir Crit Care Med.

[16] Embil J, Warren P, Yakrus M, Stark R, Corne S, Forrest D (1997). Pulmonary illness associated with exposure to Mycobacterium-avium complex in hot tub water. Hypersensitivity pneumonitis or infection?. Chest.

[17] Hilberg O, Hoffmann-Vold A-M, Smith V, Bouros D, Kilpelainen M, Guiot J, et al. Epidemiology of ILDs and their progressive-fibrosing behaviour in six European countries. ERJ Open Research [Internet]. European Respiratory Society; 2021 [cited 2022 Jan 20]; Available from: https://openres.ersjournals.com/content/early/2021/11/11/23120541.00597-2021. 10.1183/23120541.00597-2021 PMC8784757 35083316

[18] Papiris SA, Triantafillidou C, Manali ED, Kolilekas L, Baou K, Kagouridis K (2013). Combined pulmonary fibrosis and emphysema. Exp Rev Respir Med..

[19] Jankowich MD, Rounds SIS (2012). Combined pulmonary fibrosis and emphysema syndrome: a review. Chest Am Coll Chest Phys.

[20] Zantah M, Dotan Y, Dass C, Zhao H, Marchetti N, Criner GJ (2020). Acute exacerbations of COPD versus IPF in patients with combined pulmonary fibrosis and emphysema. Respir Res.

[21] Inoue Y, Kaner RJ, Guiot J, Maher TM, Tomassetti S, Moiseev S (2020). Diagnostic and prognostic biomarkers for chronic fibrosing interstitial lung diseases with a progressive phenotype. Chest.

[22] Kitaguchi Y, Fujimoto K, Hanaoka M, Honda T, Hotta J, Hirayama J (2014). Pulmonary function impairment in patients with combined pulmonary fibrosis and emphysema with and without airflow obstruction. Int J Chron Obstruct Pulmon Dis.

[23] Hansell DM, Milne DG, Wilsher ML, Wells AU (1998). Pulmonary sarcoidosis: morphologic associations of airflow obstruction at thin-section CT. Radiology.

[24] Laohaburanakit P, Chan A (2003). Obstructive sarcoidosis. Clin Rev Allergy Immunol.

[25] Ryu JH, Colby TV, Hartman TE, Vassallo R (2001). Smoking-related interstitial lung diseases: a concise review. Eur Respir J.

[26] Cigarette smoking and health. American Thoracic Society. Am J Respir Crit Care Med. 1996;153:861–5. 10.1164/ajrccm.153.2.8564146 8564146

[27] Stockley RA, Mannino D, Barnes PJ (2009). Burden and pathogenesis of chronic obstructive pulmonary disease. Proc Am Thorac Soc.

[28] Vogelmeier CF, Criner GJ, Martinez FJ, Anzueto A, Barnes PJ, Bourbeau J (2017). Global strategy for the diagnosis, management, and prevention of chronic obstructive lung disease 2017 report. Am J Respir Crit Care Med Am Thorac Soc.

[29] Pauwels RA, Buist AS, Calverley PM, Jenkins CR, Hurd SS, GOLD Scientific Committee (2001). Global strategy for the diagnosis, management, and prevention of chronic obstructive pulmonary disease. NHLBI/WHO Global Initiative for Chronic Obstructive Lung Disease (GOLD) Workshop summary. Am J Respir Crit Care Med.

[30] Han MK, Agusti A, Celli BR, Criner GJ, Halpin DMG, Roche N (2021). From GOLD 0 to Pre-COPD. Am J Respir Crit Care Med Am Thorac Soc AJRCCM.

[31] Guiot J, Vaidyanathan A, Deprez L, Zerka F, Danthine D, Frix A-N (2022). A review in radiomics: making personalized medicine a reality via routine imaging. Med Res Rev.

[32] Frix A-N, Cousin F, Refaee T, Bottari F, Vaidyanathan A, Desir C (2021). Radiomics in lung diseases imaging: state-of-the-art for clinicians. J Pers Med.

[33] Raghu G, Remy-Jardin M, Myers JL, Richeldi L, Ryerson CJ, Lederer DJ, et al. American thoracic society documents Diagnosis of Idiopathic Pulmonary Fibrosis An Official ATS/ERS/JRS/ALAT Clinical Practice Guideline. Japanese Respiratory Society. ATS; 2018.

[34] Saku A, Fujisawa T, Nishimoto K, Yoshimura K, Hozumi H, Karayama M (2021). Prognostic significance of peripheral blood monocyte and neutrophil counts in rheumatoid arthritis-associated interstitial lung disease. Respir Med.

[35] Wells AU, Flaherty KR, Brown KK, Inoue Y, Devaraj A, Richeldi L (2020). Nintedanib in patients with progressive fibrosing interstitial lung diseases—subgroup analyses by interstitial lung disease diagnosis in the INBUILD trial: a randomised, double-blind, placebo-controlled, parallel-group trial. Lancet Respir Med Lancet Publish Group.

[36] Guiot J, Cambier M, Boeckx A, Henket M, Nivelles O, Gester F (2020). Macrophage-derived exosomes attenuate fibrosis in airway epithelial cells through delivery of antifibrotic miR-142-3p. Thorax.

[37] Gribbin J, Hubbard RB, Le Jeune I, Smith CJP, West J, Tata LJ (2006). Incidence and mortality of idiopathic pulmonary fibrosis and sarcoidosis in the UK. Thorax.

[38] Wilcox AG (2000). Small airway involvement in interstitial lung disease: radiologic evidence. Curr Opin Pulmon Med..

[39] Dines DE, Stubbs SE, McDougall JC (1978). Obstructive disease of the airways associated with stage I sarcoidosis. Mayo Clin Proc.

[40] Shaw M, Collins BF, Ho LA, Raghu G (2015). Rheumatoid arthritis-associated lung disease. Eur Respir Rev Eur Respir Soc..

[41] Saag KG, Cerhan JR, Kolluri S, Ohashi K, Hunninghake GW, Schwartz DA (1997). Cigarette smoking and rheumatoid arthritis severity. Annal Rheumat Dis..

