# Supplementary material for: Combined obstructive airflow limitation associated with interstitial lung diseases (O-ILD): the bad phenotype ?
Source: Respir Res. 2022 Apr 11;23:89. doi: 10.1186/s12931-022-02006-9 (PMC8996531; doi:10.1186/s12931-022-02006-9)
Supplement: Supplementary file 1 — Additional file 1: Table S1. Evaluation of demographic and blood characteristics in each disease. [file 12931_2022_2006_MOESM1_ESM.pdf]

Additional file 1: Table S1. Evaluation of demographic and blood characteristics in each disease

	IIP		CTD		Other-ILD		Sarcoidosis		IPF	
	**Non O-IIP** (n=79)	**O-IIP** (n=12)	**Non O-CTD** (n=164)	**O-CTD** (n=22)	**Non O-other-ILD** (n=49)	**O-other-ILD** (n=14)	**Non O-sarcoid.** (n=120)	**O-sarcoid.** (n=29)	**Non O-IPF** (n=63)	**O-IPF** (n=5)
**DEMOGRAPHY**										
Age (years)	67 (58-74)	64 (59-70)	63 (52-71)	68 (62-76)	62 (56-71)	60 (47-72)	48 (38-56)	50 (43-60)	74 (68-80)	70 (66-79)
Gender (M/F)	42/37	6/6	68/96	12/10	29/20	8/6	65/55	20/9	15/48	5/0
BMI (Kg/m²)	27 (25-31)	24 (22-27)	25 (23-29)	25 (23-30)	26 (23-30)	25 (21-28)	27 (24-32)	25 (22-29)	26 (24-30)	24 (24-25)
Smokers NS/FS/CS(%)	33/30/15 N= 78 51%/46%/23%	6/4/2 50%/33%/17%	75/66/18 N=159 19%/16%/4%	6/13/3 14%/59%/27%	15/27/6 N= 48 31%/56%/13%	5/7/2 36%/50%/14%	42/7/7 N=56 75%/13%/13%	3/8/1 N= 12 25%/67%/8%	12/46/5 19%/73%/8%	0/5/0 0%/100%/0%
Delay from Onset, Months	45 (23-71)	112 (66-128)	62 (31-91)	49 (37-99)	44 (26-64)	53 (35-101)	82 (57-115)	91 (77-132)	34 (17-56)	33 (24-61)
Death ,yes (%)	21/58 (27%)	4/8 (33%)	36/128 (22%)	12/10 (55%)	13/36 (27%)	3/11 (21%)	10/110 N= 110 !!! (8%)	2/27 (7%)	39/24 (62%)	4/1 (80%)
**BLOOD ANALYSIS**										
Leucocyte (x10e3/µl)	8.97 (6.46-10.99)	8.8 (7.62-13.25)	8.25 (6.34-9.98)	9.47 (8.22-12.03)	8.12 (6.27-10.04)	8.22 (5.37-9.48)	6.52 (5.2-8.89)	7.01 (5.61-8.14)	8.1 (7.08-9.94)	10 (10-14)
Neutrophil (%)	63 (55-76)	66 (62-71)	66 (60-76)	72 (65-79)	62 (57-73)	66 (55-78)	66 (58-71)	73 (61-76)	64 (55-71)	73 (55-96)
(Cell/mm³)	5.4 (3.82-7.45)	5.08 (4.91-8.33)	5.37 (3.81-6.84)	6.03 (5.56-8.79)	4.95 (3.7-6.82)	5.49 (3.41-6.93)	4.37 (3.3-5.89)	5.02 (3.72-5.64)	5.04 (4.33-6.64)	7.58 (5.38-13.18)
Lymphocyte (%)	24 (15-33)	22 (19-27)	22 (14-28)	19 (11-24)	23 (16-30)	19 (12-23)	21 (16-27)	16 (13-26)	23 (16-31)	15 (2-24)
(Cell/mm³)	1.96 (1.34-2.55)	2.02 (1.3-2.95)	1.51 (1.16-2.1)	1.93 (1.2-2.29)	1.73 (1.29-2.24)	1.49 (1.03-1.86)	1.33 (1-1.88)	1.09 (0.85-2.01)	1.9 (1.31-2.33)	1.54 (0.2-2.37)
Monocyte (%)	7.35 (5.65-9.4)	7.5 (4.8-8.6)	7.35 (5.7-9.2)	7.3 (4.8-8.7)	6.8 (5.2-10)	6.4 (5.2-8.45)	8.5 (6.3-10.8)	9.5 (6-10.8)	7.9 (6.7-10.4)	8.5 (1.7-10.9)
(Cell/mm³)	0.63 (0.42-0.94)	0.49 (0.37-0.8)	0.59 (0.45-0.77)	0.61 (0.5-0.92)	0.55 (0.48-0.69)	0.49 (0.37-0.71)	0.58 (0.42-0.73)	0.61 (0.47-0.76)	0.66 (0.47-0.87)	0.83 (0.24-1.13)
Eosinophil (%)	1.95 (1.1-3.1)	2 (1.6-2.3)	2.1 (1.1-3.3)	1.6 (0.5-2.6)	2.2 (1.5-3.9)	3.6 (0.45-5.8)	2.5 (1.4-3.9)	1.9 (1.2-2.8)	3.5 (2-5.4)	1.2 (0.6-11.1)
(Cell/mm³)	0.17 (0.06-0.29)	0.15 (0.12-0.21)	0.16 (0.09-0.26)	0.18 (0.05-0.22)	0.19 (0.13-0.3)	0.19 (0.05-0.41)	0.16 (0.09-0.26)	0.15 (0.09-0.25)	0.29 (0.15-0.43)	0.13 (0.08-1.08)
Basophil (%)	0.4 (0.2-0.65)	0.5 (0.3-0.5)	0.4 (0.3-0.6)	0.3 (0.2-0.4)	0.4 (0.2-0.7)	0.45 (0.15-1.05)	0.5 (0.3-0.7)	0.5 (0.3-0.7)	0.6 (0.3-0.8)	0.3 (0.1-0.7)
(Cell/mm³)	0.03 (0.02-0.06)	0.04 (0.02-0.05)	0.03 (0.02-0.05)	0.03 (0.02-0.04)	0.03 (0.02-0.06)	0.03 (0.02-0.06)	0.03 (0.02-0.05)	0.04 (0.03-0.05)	0.05 (0.02-0.06)	0.03 (0.02-0.07)
CRP (mg/L)	7.86 (2.25-35.99)	9.85 (5.5-15.48)	4.08 (1.58-15.12)	14 (3-32)	8.09 (2.47-26)	4.71 (1.6-12.03)	4.14 (2-13)	4.52 (2.71-37.3)	5.43 (2.58-14.71)	23 (2-27)
Fibrinogen (g/L)	4.03 (3.34-4.65)	3.32 (2.69-4.46)	4.02 (3.38-5.08)	4.64 (3.64-5.86)	4.07 (3.15-5.43)	3.85 (3.08-4.97)	3.53 (2.97-4.47)	3.78 (3.05-5.82)	3.51 (2.79-4.72)	3.56 (3.41-3.76)
GFR (ml/min/1.73m²)	79 (63-98)	88 (71-93)	80 (66-94)	75 (58-87)	72 (46-90)	77 (45-98)	84 (70-97)	85 (72-97)	70 (55-83)	73 (70-97)

Data are expressed as median (IQR). Data are analysed using a two-tailed Mann-Whitney test, followed by Bonferroni correction. BMI = Body mass index; CTD = connective tissue disease; GFR = glomerular filtration rate; CRP = C-reactive protein; IIP = idiopathic interstitial pneumonia; ILD = Interstitial lung disease; IPF = idiopathic pulmonary fibrosis; NS/FS/CS = non smokers/former smokers/current smokers; O- = obstructive-.
