# Supplementary material for: Combined obstructive airflow limitation associated with interstitial lung diseases (O-ILD): the bad phenotype ?
Source: Respir Res. 2022 Apr 11;23:89. doi: 10.1186/s12931-022-02006-9 (PMC8996531; doi:10.1186/s12931-022-02006-9)
Supplement: Supplementary file 2 — Additional file 2: Table S2. Evaluation of pulmonary functional tests in each disease. [file 12931_2022_2006_MOESM2_ESM.pdf]

Additional file 2: Table S2. Evaluation of pulmonary functional tests in each disease

	IIP		CTD		Other ILD		Sarcoidosis		IPF	
	**Non O-IIP** (n=79)	**O-IIP** (n=12)	**Non O-CTD** (n=164)	**O-CTD** (n=22)	**Non O-other-ILD** (n=49)	**O-other-ILD** (n=14)	**Non O-sarcoid.** (n=120)	**O-sarcoid.** (n=29)	**Non O-IPF** (n=63)	**O-IPF** (n=5)
**PULMONARY FUNCTIONAL TEST**										
**FEV1 (L)**	2.15 (1.68-2.62)	1.78 (1.29-2.41)	2.21 (1.71-2.74)	1.68 (1.42-2.31)	2.37 (1.66-2.8)	2.04 (1.51-2.59)	2.77 (2.12-3.49)	1.82 (1.29-2.48)	1.99 (1.67-2.56)	1.8 (1.53-2.51)
**FEV1 (%pred.)**	84 (68-97)	62 (56-77)	84 (69-97)	71 (62-77)	82 (70-89)	77 (65-81)	85 (75-97)	55 (42-67)*	78 (71-94)	60 (57-81)
**FVC (L)**	2.58 (2.04-3.26)	3.35 (2-4.05)	2.71 (2.1-3.45)	2.79 (2.28-3.44)	3.07 (2.18-3.64)	3.28 (2.87-3.9)	3.43 (2.62-4.41)	3.25 (2.18-4.13)*	2.46 (2.02-3.21)	2.91 (2.4-3.71)
**FVC , %pred.**	81 (67-94)	84 (80-97)	84 (68-98)	89 (77-99)	83 (68-90)	100 (80-104)	87 (78-98)	79 (67-88)	75 (63-88)	71 (69-92)
**FEV1/FVC post-BD, %pred.**	83 (79-89)	63 (59-65)*	82 (77-86)	63 (59-67)*	79 (77-85)	61 (59-67)*	81 (76-85)	60 (53-65)	82 (78-86)	68 (64-68)
**DEM25/75 (L)**	2.5 (1.83-3.22)	0.75 (0.47-1.07)*	2.43 (1.89-2.92)	1.27 (1.17-1.37)	2.31 (1.55-2.77)	1.13 (0.81-1.48)*	2.71 (2.18-3.44)	1.03 (0.53-1.35)*	2.4 (1.64-3.28)	0.83 (0.81-1.49)
**DEM25/75 (%)**	94 (70-119)	25 (18-32)*	80 (61-104)	38 (35-40)	77 (59-104)	40 (29-49)*	73 (62-87)	22 (15-34)*	95 (64-143)	32 (26-46)
**RV (L)**	1.76 (1.25-2.26)	2.98 (2.65-3.09)	1.86 (1.4-2.36)	2.43 (1.75-3.43)	1.93 (1.49-2.38)	2.81 (2.25-3.26)*	1.61 (1.28-1.97)	2.15 (1.75-2.87)*	1.53 (1.34-1.95)	1.87 (1.78-2.43)
**RV (%pred.)**	79 (60-102)	130 (115-164)	88 (70-110)	107 (80-133)	87 (68-108)	125 (95-165)*	85 (68-102)	119 (80-148)	64 (52-82)	71 (68-92)
**TLC (L)**	4.37 (3.48-5.2)	6.31 (5.56-7.13)	4.6 (3.67-5.43)	5.18 (4.48-6.37)	4.87 (4.32-5.52)	5.96 (5.75-6.17)	5.21 (4.08-6.04)	5.64 (4.43-6.64)	4.17 (3.25-5.01)	5.57 (4.48-5.73)
**TLC (%pred.)**	75 (65-88)	103 (98-114)	79 (70-96)	90 (79-107)	80 (71-91)	98 (89-107)*	86 (75-94)	92 (80-103)	70 (60-77)	78 (68-83)
**DLco (mmol/kPa.min)**	3.7 (2.69-4.96)	4.35 (3.3-5.3)	4.01 (2.2-5.7)	0.77 (0.33-3.24)	4.52 (3.32-6.68)	5.55 (3.6-6.4)	0.94 (0.54-6.3)	0.71 (0.48-0.85)	3.59 (2.49-4.47)	2.6 (2.4-4.18)
**DLco (%pred.)**	47 (36-66)	46 (41-62)	57 (41-73)	52 (36-69)	56 (42-73)	65 (49-68)	71 (56-85)	63 (50-74)	44 (32-55)	37 (28-46)
**KCO (mmol/kPa.min/L)**	1.06 (0.84-1.33)	1.15 (0.92-1.27)	1.16 (0.94-1.37)	1.01 (0.84-1.05)	1.19 (0.94-1.38)	1.13 (0.81-1.27)	1.47 (1.3-1.71)	1.39 (1.14-1.65)	0.94 (0.79-1.13)	0.74 (0.7-0.93)
**KCO (%pred.)**	73 (63-92)	81 (54-95)	79 (66-92)	70 (62-82)	84 (66-99)	75 (63-87)	94 (79-110)	92 (74-102)	75 (58-88)	58 (53-69)
**FRC (L)**	2.62 (2.22-3.45)	3.77 (3.43-4.68)	2.75 (2.36-3.63)	3.15 (2.66-5.69)	3.13 (2.72-3.73)	3.46 (3.27-4.64)	2.72 (2.38-3.43)	3.49 (2.9-4.42)	2.7 (2.15-3.25)	3 (2.25-4.02)
**FRC (%pred.)**	88 (71-109)	127 (106-140)	97 (75-115)	105 (90-155)	97 (81-113)	117 (94-123)	89 (75-109)	111 (96-121)	83 (66-95)	82 (66-103)
**sGaw (L)**	1.05 (0.76-1.4)	0.59 (0.48-1.14)	1.16 (0.79-1.68)	0.81 (0.61-0.98)	1.02 (0.76-1.27)	0.61 (0.47-0.68)	1.22 (0.82-1.57)	0.74 (0.44-0.84)	1.23 (0.95-1.64)	1.19 (0.87-1.41)
**sGaw (%)**	80 (50-121)	112 (112-112)	91 (60-116)	60 (49-77)	88 (60-121)	42 (35-62)	77 (54-115)	64 (36-75)*	95 (71-131)	174 (174-174)

*Data are expressed as median (IQR). * P<0.05 compared to non O-ILD subgroup. Data are analysed using a two-tailed Mann-Whitney test, followed by Bonferroni correction. CRP = C-reactive protein; CTD = connective tissue disease; DLCO = Diffusing lung capacity of CO; FEV1 = Forced expired volume in 1 second; FRC = Functional residual capacity; FVC =Forced vital capacity; GFR = glomerular filtration rate; IIP = idiopathic interstitial pneumonia; ILD = Interstitial lung disease; IPF = idiopathic pulmonary fibrosis; KCO: DLCO/Alveola ventilation; MEF = Maximum expiratory flow; NS/FS/CS = non smokers/former smokers/current smokers; O- = obstructive-; RV: Residual volume; TLC = Total lung capacity; sGaw = specific conductances.*
