# Supplementary material for: Combined obstructive airflow limitation associated with interstitial lung diseases (O-ILD): the bad phenotype ?
Source: Respir Res. 2022 Apr 11;23:89. doi: 10.1186/s12931-022-02006-9 (PMC8996531; doi:10.1186/s12931-022-02006-9)
Supplement: Supplementary file 3 — Additional file 3: Table S3. Evolution of pulmonary functional test over 3 years. [file 12931_2022_2006_MOESM3_ESM.pdf]

Additional file 3: Table S3. Evolution of pulmonary functional test over 3 years

	Non O-ILD	O-ILD
**Delay V1 - V2 (years)**	2.89 (2.47-3.16)	2.78 (2.3-3.05)
**Δ FEV1 (%)**	-3.26 (-11.89-4.79)	-5.71 (-15.23-10.22)
**Δ FEV1 (%/year)**	-1.12 (-4.35-1.85)	-2.06 (-5.69-3.45)
**Δ FEV1 (ml)**	-80 (-280-120)	-90 (-300-100)
**Δ FEV1 (ml/year)**	-31.85 (-104.36-42.61)	-32.28 (-137.71-61.98)
**Δ FVC (%)**	-2.01 (-10.05-5.76)	-5.21 (-16.08-9.37)
**Δ FVC (%/year)**	-0.73 (-3.81-2.10)	-1.78 (-5.19-3.49)
**Δ FVC (ml)**	-60 (-300-140)	-110 (-410-190)
**Δ FVC (ml/year)**	-25.54 (-111.02-55.51)	-40.14 (-183.37-80.89)
**Δ DLco (%)**	-5.77 (-24.73-9.78)	-12.17 (-26.09-3.85)
**Δ DLco (%/year)**	-2.34 (-9.34-3.55)	-3.98 (-9.76-1.34)
**Δ DLco (mmol/kPa.min)**	-200 (-1060-520)	-400 (-940-200)
**Δ DLco (mmol/kPa.min/year)**	-89.3 (-387.39-201.81)	-175.14 (-381.25-113.62)

DLco : Diffusing lung capacity of CO; FEV1: Forced expired volume in 1 second; FVC: Forced vital capacity; ILD = interstitial lung disease; O-ILD = obstructive- interstitial lung disease.
