# Supplementary material for: Combined obstructive airflow limitation associated with interstitial lung diseases (O-ILD): the bad phenotype ?
Source: Respir Res. 2022 Apr 11;23:89. doi: 10.1186/s12931-022-02006-9 (PMC8996531; doi:10.1186/s12931-022-02006-9)
Supplement: Supplementary file 4 — Additional file 4: Fig. S1. Evolution of pulmonary functional test over 3 years from the last visit. [file 12931_2022_2006_MOESM4_ESM.pdf]

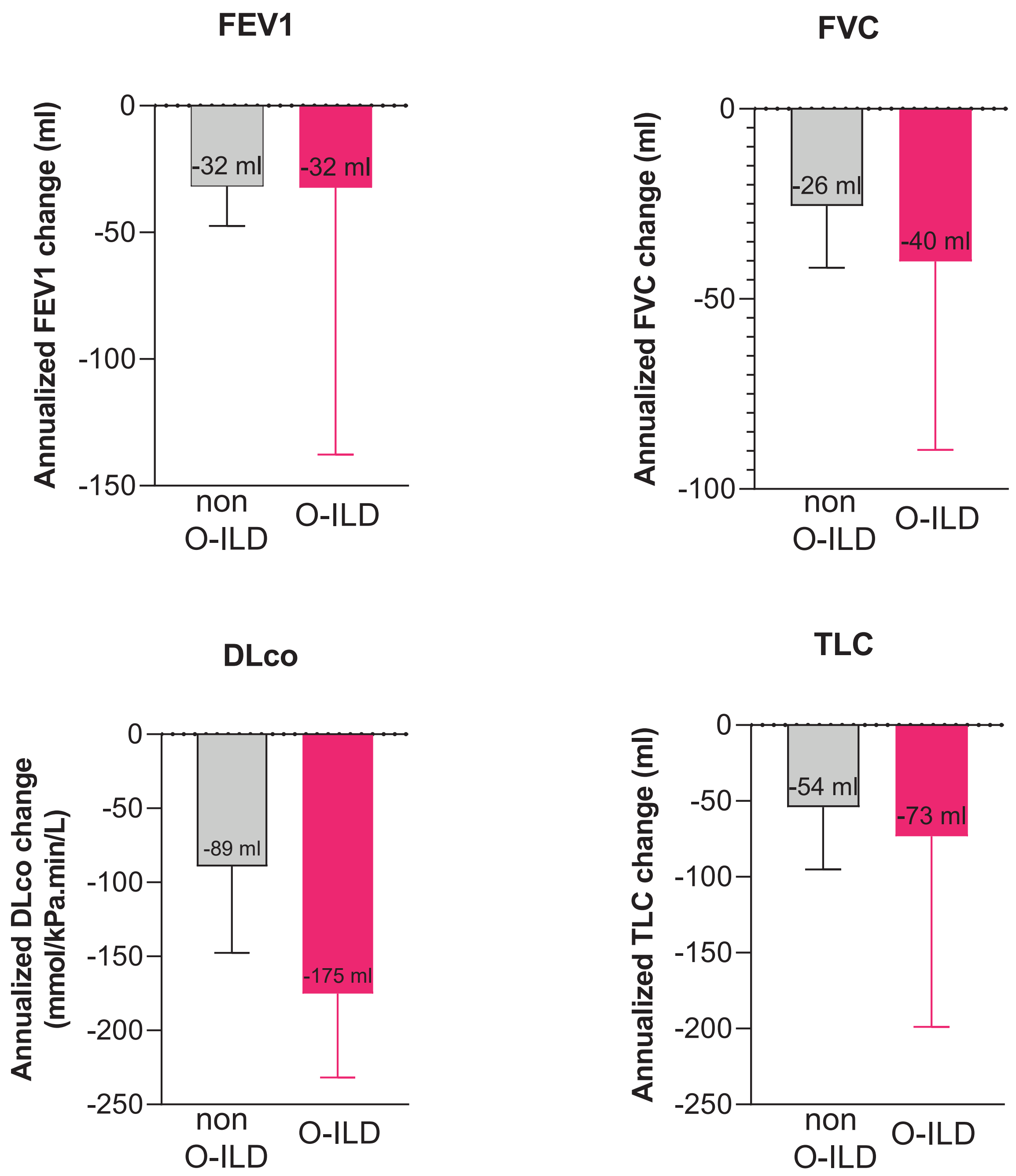

Additional file 4: Fig. S1: Evolution of pulmonary functional test over 3 years from the last visit. Bar represents Median value with 95% CI.
DLco = Diffusing lung capacity of CO; FEV1 = Forced expired volume in 1 second;
FVC = Forced vital capacity; TLC =Total lung capacity.
